# Prevalence and predictors of functional gastrointestinal disorder among the undergraduate students of Bangladesh

**DOI:** 10.1371/journal.pone.0315687

**Published:** 2024-12-18

**Authors:** Simanta Roy, Fahima Nasrin Eva, Dipa Dev, Sanchita Roy, Shafkat Kamal Tipu, Sristi Chowdhury, Madhu Ritu Bhadra Medha, Purzia Tanaz Haque Poonya, Israt Jahan Juthi, Jwearia Hoque Nowrin, Eaasvar J. C., Tahsin Sumat, Disha Mony Dey, Sreshtha Chowdhury, Mohammad Azmain Iktidar, Mohammad Delwer Hossain Hawlader

**Affiliations:** 1 Department of Public Health, North South University, Dhaka, Bangladesh; 2 School of Research, Chattogram, Bangladesh; 3 Public Health Promotion and Development Society (PPDS), Dhaka, Bangladesh; 4 Department of Medicine, Chittagong Medical College Hospital, Chittagong, Bangladesh; 5 Rangpur Medical College, Rangpur, Bangladesh; 6 Department of Biochemistry and Molecular Biology, Noakhali Science and Technology University, Noakhali, Bangladesh; 7 Department of Medicine, Shaheed M. Monsur Ali Medical College, Sirajganj, Bangladesh; 8 Centre for Injury Prevention and Research, Dhaka, Bangladesh; 9 Department of Medicine, Dhaka National Medical College, Dhaka, Bangladesh; 10 Department of Medicine, Cumilla Medical College Hospital, Cumilla, Bangladesh; 11 Department of Medicine, M Abdur Rahim Medical College, Dinajpur, Bangladesh; Quaid-i-Azam University Islamabad: Quaid-i-Azam University, PAKISTAN

## Abstract

**Objective:**

This study aimed to address this knowledge gap by investigating FGID prevalence and its predictors among undergraduate students in Bangladesh.

**Design:**

This cross-sectional study was conducted between 01 August 2023 and 31 January 2024 among 1,019 undergraduate students. Data were collected using a web-based survey containing questions on socio-demographics, the Rome IV questionnaire, the insomnia severity index, the perceived stress scale 4, the patient health questionnaire, and the smartphone addiction scale. Descriptive statistics, the chi-square test, the t-test, and the multivariable logistic regression model were used to report our study findings.

**Results:**

The overall prevalence of FGID was 38.24%, with functional constipation being the most common subtype (18.24%). The multivariate analysis revealed that college canteen meal (AOR: 1.593, CI: 1.068, 2.376), occasionally and regularly delayed meal (AOR: 1.663, CI: 1.031, 2.682; AOR: 1.872, CI: 1.061, 3.301), physical inactivity (AOR:0.41, CI: 1.061, 3.301), family history of FGID and GI disease (AOR: 4.7, CI: 2.55, 8.66; AOR: 2.42, CI: 1.47, 3.96), history of abdominal surgery (AOR: 2, CI: 1.08, 3.72), psychological trauma (AOR: 1.64, CI: 1.04, 2.57), dairy-product consumption (AOR: 1.64, CI: 1.04, 2.59), >3 meals/day (AOR: 1.89, CI: 1.2, 2.98), insomnia (AOR: 1.98, CI: 0.73, 5.40), and depression (AOR: 7.02, CI: 2.74, 17.98) were significantly associated with FGID.

**Conclusion:**

The burden of FGIDs among Bangladeshi students is concerning. This study found significant factors contributing to their prevalence, including meal source and number of daily meals, delayed meals, family history of disease, physical activity, abdominal surgery, history of psychological trauma, depression, and insomnia. This study recommends further exploration and holistic healthcare approaches to better the well-being of young adults dealing with FGIDs.

## Introduction

Functional gastrointestinal disorders (FGIDs) encompass a set of conditions characterized by gastrointestinal (GI) symptoms attributed to various factors, including motility disturbance, visceral hypersensitivity, altered mucosal and immune function, changes in gut microbiota, and modified central nervous system processing [1]. Despite not posing an increased risk of mortality, FGIDs contribute significantly to morbidity, negatively impacting the quality of life and leading to a heightened reliance on healthcare resources [1,2].

FGIDs are widespread, affecting 40% of the global population, with higher prevalence in women and a tendency to decrease with age [3]. These disorders constitute 12% of primary care cases and contribute to 30% of gastroenterology outpatient consultations [4,5]. However, only a few studies have assessed the overall FGID prevalence and prevalence of all FGID subtypes in a particular adult population [6,7]. The most studied FGIDs, functional dyspepsia (FD) and irritable bowel syndrome (IBS) [8], show varying prevalence globally. The prevalence of FD ranges from 23% to 26% in the West and 12% to 29% in the East [2,9] and for IBS, 9% to 29.2% in the West and 3.5% to 14.2% in the East based on Rome II/III criteria [10–12]. A recent survey using Rome IV criteria in the West found prevalence rates for IBS, functional constipation (FC), functional diarrhoea (FDr), and functional bloating at 4.4% to 4.8%, 7.9% to 8.6%, 3.6% to 5.3%, and 2.0% to 3.9%, respectively [13].

Several studies assessed the factors associated with FGIDs among diverse groups of populations. In one prior study, female gender, medical student status, non-vegetarian diet, junk food, tea, coffee, insufficient physical exercise, anxiety, and sleeplessness were found to be independent predictors for FGIDs [14]. Also, substantial evidence shows the relationship between psychological factors and FGIDs [15]. Moreover, BMI, social media, and ultra-processed food consumption were also found as potential factors of FGID in prior studies [16–18].

Undergraduate students often contend with distinct socioeconomic and psychological challenges, heightening their susceptibility to Functional Gastrointestinal Disorders (FGIDs) [19]. The rising prevalence of lower physical activity, increasing BMI, unhealthy dietary practices, mental health conditions, and increasing use of digestive medication, analgesics, and alternative medicine acknowledges the need for further examining the factors of FGIDs among the younger population [20–24]. With over 4 million undergraduate students in Bangladesh [25], a significant majority residing in dorms or mess halls, the challenge of maintaining a healthy lifestyle amplifies vulnerability to FGIDs [26,27]. Despite this, data on the prevalence of all types of FGIDs in Bangladesh are limited. Notably, prior studies in Bangladesh predominantly concentrate on investigating irritable bowel syndrome (IBS) [28,29], disregarding a comprehensive exploration of other FGIDs among specific populations, such as undergraduate students.

Addressing FGIDs in undergraduate students has significant public health and policy implications. First, increasing awareness about FGIDs in this population can lead to earlier detection and better management, reducing the long-term health and economic burden. Policy initiatives should promote healthier dietary and lifestyle practices within university settings, including improved food quality in dormitories and increased access to mental health resources to mitigate stress-related GI symptoms. Furthermore, enhancing medical education on FGIDs among healthcare providers could improve the diagnosis and treatment of these disorders. Therefore, this study, by assessing the prevalence and predictors of FGIDs among Bangladeshi undergraduate students, fills a crucial research gap and provides evidence that can guide policy formulation and health promotion strategies aimed at reducing the burden of FGIDs in this vulnerable population.

## Methods

### Study population and sampling

This cross-sectional study was carried out among undergraduate students of Bangladesh between August 2023 and January 2024. A total of 1,019 undergraduate students participated in this study. Students with diagnosed organic gastrointestinal disorders were excluded. Trained research assistants contacted prospective participants via snowball sampling (using phone calls/social media) and described the research in detail. Once the individuals were ascertained to meet the inclusion criteria and consented to voluntary participation (verbally) in the study, a link to a web-based survey created by Google Forms was sent via Facebook messenger/email/SMS, making it a closed survey. The survey wasn’t announced or advertised anywhere else. Of the 1,035 eligible participants who agreed to participate, 1,019 completed the entire questionnaire (completion rate: 99.4%); incomplete questionnaires were excluded from the analysis.

### Study instruments

We collected data from the study participants using a structured online questionnaire. Participants’ socio-demographic information, such as gender, age, educational institution, department, marital status, monthly family income in BDT, and living place, was collected. Additionally, participants provided details on disease history (personal and family), deworming practices, abdominal surgery history, history of psychological trauma, and lifestyle-related factors, including delayed meals, smoking habits, physical activity, frequency of fast-food intake, dairy product consumption, caffeine intake, and number of meals per day. The study participants’ BMI was calculated using their weight in kilograms and height in feet and inches. The standard BMI formula was applied, involving the conversion of height to meters and the division of weight by the squared height.

The Insomnia Severity Index (ISI) was employed to evaluate insomnia among the participants. ISI is a self-report questionnaire consisting of seven items designed to assess the nature, severity, and impact of insomnia [30,31]. It typically covers the "last month" as the recall period and evaluates dimensions such as the severity of sleep onset, sleep maintenance, and early morning awakening problems, sleep dissatisfaction, interference of sleep difficulties with daytime functioning, noticeability of sleep problems by others, and distress caused by the sleep difficulties. Each item is rated on a 5-point Likert scale (e.g., 0 = no problem; 4 = very severe problem), resulting in a total score ranging from 0 to 28. The total score is interpreted as follows: absence of insomnia (0–7); sub-threshold insomnia (8–14); moderate insomnia (15–21); and severe insomnia (22–28).

Previously validated Perceived Stress Scale 4 (PSS-4) [32], Patient Health Questionnaire (PHQ-9), [33,34] and smartphone addiction scale (SAS-SV) were used to assess stress [35], depression, and level of screen addiction among the study participants, respectively.

PSS-4 is a validated 4-item tool in which respondents rated how frequently they personally experienced stress during the previous two weeks on a 5-point scale ranging from ’0’ (Never) to ’4’ (Very often). Total PSS-4 scores range from as low as 0 to as high as 16, with higher values suggesting more stress [32].

We used the validated 9-item PHQ-9 questionnaire to measure depression [33,34,36]. On a scale from ’0’ (not at all) to ’3’ (nearly every day), participants indicated how much each of the nine depression symptoms disturbed them over the previous two weeks. The sum of all item scores, which range from 0 to 27, was used to determine the overall score, with a higher score indicating greater depression. To measure the severity of depression, we used categories (1–4: None; 4–9: Mild; 10–14: Moderate; 15–19: Moderately severe; 20–27).

We used SAS-SV, which was developed by Kwon et al., to determine smartphone addiction among the study participants. This scale has six components (daily-life disturbance, positive anticipation, withdrawal, cyberspace-oriented relationship, overuse, and tolerance from the original version of the smartphone addiction scale) with ten items each that were scored using a six-point Likert scale (1 = strongly disagree, 2 = disagree, 3 = weakly disagree, 4 = weakly agree, 5 = agree, and 6 = strongly agree) [35]. The final SAS-SV score was calculated by summing the responses for each item. Based on validated cutoffs, a score of 31 or higher for males and 33 or higher for females indicated smartphone addiction [35]. To refine the analysis, we further classified participants into three distinct categories based on their SAS-SV scores:

1. Not addicted: Scores below 31 for males and 33 for females indicated no risk of smartphone addiction.
2. High risk: Participants with scores ranging from 31–39 for males and 33–39 for females were classified as being at high risk for smartphone addiction.
3. Addicted: Scores 40 and above for both males and females indicated full smartphone addiction.

The Rome IV questionnaire was utilized to investigate digestive symptoms diagnosing various types of FGIDs [37].

### Ethics

The Institutional Review Board of North South University approved the research (approval no: 2023/OR-NSU/IRB/0305). Wherever feasible, the 1964 Declaration of Helsinki and later modifications and comparable ethical standards were followed. Data collection was voluntary, and consent (verbal) was taken prior to data collection. Also, the first part of the study instrument (Google Form) provided the study information and consent statement. Participants were asked to indicate their consent to participate by checking a box before moving on to the next sections. No incentives were offered to participants. Data were only accessible to the research team and were not disclosed anywhere. All the reporting was done according to the Checklist for Reporting Results of Internet E-Surveys (CHERRIES) guidelines [38].

### Statistical analysis

The statistical software Stata (version 17.0) was used to analyze the data. We displayed the categorical variables as frequencies, and their respective percentages and means with standard deviations were shown for continuous variables. Pearson’s chi-square test evaluated the association between categorical independent variables and the outcome variables. A binary logistic regression model was fitted to identify the factors associated with FGID. The explanatory variables included in the model were chosen based on a comprehensive review of relevant literature and their theoretical relevance to FGID. Variables such as socio-demographic characteristics (age, gender), lifestyle factors (physical activity, smoking, fast food intake), and psychological factors (stress, depression, and smartphone addiction) were selected considering their strong associations with FGIDs in prior studies and bivariate analysis conducted in our current study. We employed a stepwise regression approach to determine the final model. The lowest values of the Akaike Information Criterion (AIC) and the Bayesian Information Criterion (BIC) were considered for model selection. The variance inflation factor (VIF) was used to measure the presence of multicollinearity (VIF <5 for all). A p-value of <0.05 was considered statistically significant.

## Results

**Fig 1:**
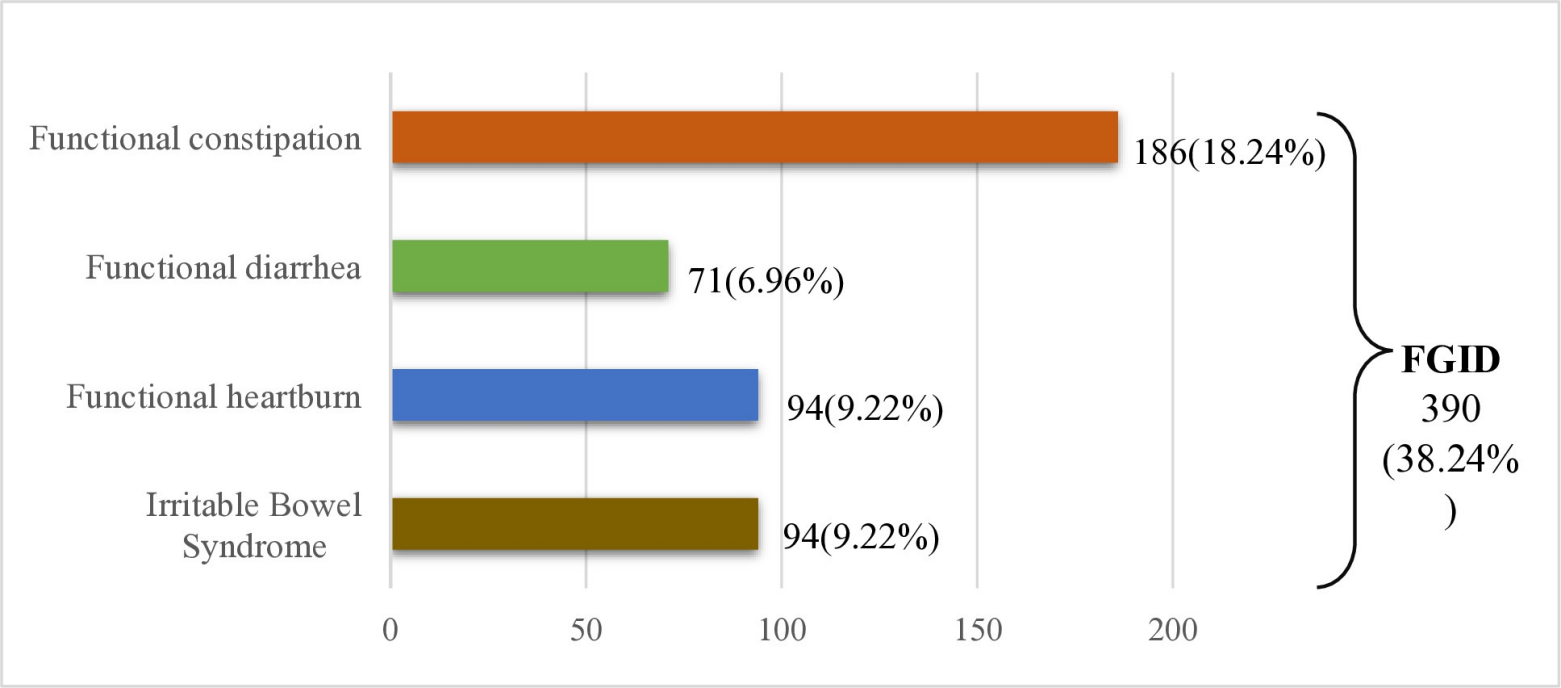
Prevalence of functional gastrointestinal disorder (N = 1,019).

This study explored the prevalence of functional gastrointestinal disorders among undergraduate students in Bangladesh (**Fig 1**). Overall, 38.24% of participants reported FGID symptoms, and the majority were suffering from functional constipation (18.24%).

A total of 1,019 undergraduate students and their demographic characteristics are shown in Table 1. The mean age of the participants was 22.36±2.47, where the majority were female (54.47%) and unmarried (93.91%). Most of them studied in public institutions (70.) and lived in hostels (50.05%). Most reported occasional delayed meals (67.12%) and fast food, dairy products, and caffeine intake 1–3 times per week (67.03%, 56.13% & 73.50% respectively). They were moderately active in their daily lives (65.) with a normal BMI (59.46%). Family history of FGIDs was found in 7.66%, whereas a history of abdominal surgery and psychological trauma were reported among 5.69% and 11.19%, respectively. Most of them used to have three meals per day (52.99%). Subthreshold insomnia and moderate depression were found in 48.48%, 33.37%, and 82.04% were highly addicted to screens.

**Table 1:** Baseline characteristics of the participant and their relation to functional gastrointestinal disorder (N = 1,019).

		FGID		
Variables		No	Yes	p value
	N (%)	N (%)	N (%)	
**Age (mean±SD)**	22.26±2.47	22.36**±**2.64	22.09**±**2.16	0.140^#^
**Gender**				0.103
Female	555(54.47)	330(59.46)	225(40.54)	
Male	464(45.53)	299(64.44)	165(35.56)	
**Institution**				0.562
Public	723(70.95)	440(60.86)	283(39.14)	
Private	267(26.20)	169(63.30)	98(36.70)	
Others	29(2.85)	20(68.97)	9(31.03)	
**Department**				0.955
Medical	504(49.46)	309(61.31)	195(38.69)	
Biological science	62(6.08)	39(62.03)	23(37.10)	
Others	453(44.46)	281(62.03)	172(37.97)	
**Marital status**				0.312
Unmarried	956(93.91)	586(61.30)	370(38.70)	
Married	62(6.09)	42(67.74)	20(32.26)	
**Income (in BDT)**				0.076
<10000	88(8.64)	44(50.00)	44(50.00)	
10000–30000	293(28.75)	184(62.80)	109(37.20)	
30001–60000	371(36.41)	240(64.69)	131(35.31)	
>60000	267(26.20)	161(60.30)	106(39.70)	
**Living place**				0.572
Home (with family)	480(47.11)	303(63.13)	177(36.88)	
Home (Relatives)	29(2.85)	16(55.17)	13(44.83)	
Hostel	510(50.05)	310(60.78)	200(39.22)	
**Delayed meal**				**0.012** *
Never	143(13.25)	101(70.63)	42(29.37)	
Occasionally	684(67.12)	423(61.84)	261(38.16)	
Regularly	192(18.84)	105(54.69)	87(45.31)	
**Smoking**				0.371
Regular smoker	66(6.48)	38(57.58)	28(42.42)	
Former smoker	22(2.16)	10(45.45)	12(54.55)	
Never smoker	889(87.24)	555(62.43)	334(37.57)	
Occasional smoker	42(4.12)	26(61.90)	16(38.10)	
**Physical activity**				**<0.001** *
Inactive	197(19.33)	104(52.79)	93(47.21)	
Moderately active	672(65.95)	407(60.57)	265(39.43)	
Very active	150(14.72)	118(78.67)	32(21.33)	
**Family history**				**<0.001** *
None	255(25.05)	191(74.90)	64(25.10)	
Functional gastrointestinal disease	78(7.66)	29(37.18)	49(62.82)	
Other Gastrointestinal disease	126(12.38)	67(53.17)	59(46.83)	
Other diseases	559(54.91)	342(61.18)	217(38.82)	
**Deworming**				0.203
Regularly	299(29.34)	191(63.88)	108(36.12)	
Irregularly	513(50.34)	303(59.06)	210(40.94)	
Never	207(20.31)	135(65.22)	72(34.78)	
**History of abdominal surgery**				**0.014** *
No	961(94.31)	602(62.64)	359(37.36)	
Yes	58(5.69)	27(46.55)	31(53.45)	
**History of psychological trauma**				**<0.001** *
No	905(88.81)	578(63.87)	327(36.13)	
Yes	114(11.19)	51(44.74)	63(55.26)	
**BMI**				0.652
Underweight	96(9.51)	62(64.58)	34(35.42)	
Normal	600(59.46)	372(62.00)	228(38.00)	
Overweight/Obese	313(31.02)	187(59.74)	126(40.26)	
**Fast-food intake**				0.881
Never	189(18.55)	117(61.90)	72(38.10)	
1–3 times/week	683(67.03)	424(62.08)	259(37.92)	
Daily	147(14.43)	88(59.86)	59(40.14)	
**Fruits and vegetable intake**				0.681
Never	46(4.51)	26(56.52)	20(43.48)	
1–3 times/week	654(64.18)	402(61.47)	252(38.53)	
Daily	319(31.31)	201(63.01)	118(36.99)	
**Dairy products intake**				0.083
Never	255(25.02)	161(63.14)	94(36.86)	
1–3 times/week	572(56.13)	363(63.46)	209(36.54)	
Daily	192(18.84)	105(54.69)	87(45.31)	
**Caffeine intake**				0.908
Never	115(11.29)	70(60.87)	45(39.13)	
1–3 times/week	749(73.50)	461(61.55)	288(38.45)	
Daily	155(15.21)	98(63.23)	57(36.77)	
**Number of meals per day**				0.094
≤ 2 meals/day	200(19.63)	135(67.50)	65(32.50)	
3 meals/day	540(52.99)	333(61.67)	207(38.33)	
>3 meals/day	279(27.38)	161(57.71)	118(42.29)	
**Insomnia**				**<0.001** *
No insomnia	328(32.19)	250(76.22)	78(23.78)	
Subthreshold insomnia	494(48.48)	292(59.11)	202(40.89)	
Moderately severe insomnia	176(17.27)	78(44.32)	98(55.68)	
Severe insomnia	21(2.06)	9(42.86)	12(57.14)	
**Stress score (mean±SD)**	6.13**±**2.62	5.78**±**2.56	6.70**±**2.62	**<0.001** *
**Depression**				**<0.001** *
Minimal depression	168(16.49)	143(85.12)	25(14.88)	
Mild depression	285(27.97)	189(66.32)	96(33.68)	
Moderate depression	340(33.37)	194(57.06)	146(42.94)	
Moderately severe depression	197(19.33)	92(46.70)	105(53.30)	
Severe depression	29(2.85)	11(37.93)	18(62.07)	
**Screen addiction**				0.060
Not addicted	35(3.43)	25(71.43)	10(28.57)	
High risk	148(14.52)	102(68.92)	46(31.08)	
Addicted	836(82.04)	502(60.05)	334(39.95)	

* = Significant (p<0.05).

In bivariate analysis, FGID was associated with delayed meals (p = 0.012), physical inactivity (p = 0.001), family history of FGID (p = 0.001), history of abdominal surgery (p = 0.014), history of psychological trauma (p = 0.001), insomnia (p = 0.001), stress (p = 0.001) and depression (p = 0.001).

After adjusting the variables in the multivariate analysis, odds of FGID was 1.59 times higher in those who had meals from college canteens (AOR = 1.593; CI: 1.068–2.376) than those who had home-cooked meals. Timeliness in meal intake proved significant, as participants reporting occasional delays (AOR = 1.663; CI: 1.031–2.682) or regular delays (AOR = 1.872; CI: 1.061–3.301) exhibited heightened FGID risk compared to those maintaining a consistent schedule. Physical activity is protective, as individuals with high activity levels (AOR = 0.409; CI: 0.236–0.709) are significantly less likely to experience FGID. Familial factors contributed significantly to the likelihood, with individuals having a family history of FGID (AOR = 4.697; CI = 2.547–8.662), other Gastrointestinal diseases (AOR = 2.417; CI = 1.466–3.985), and other diseases (AOR = 1.943; CI = 1.337–2.823) facing increased odds. Previous abdominal surgery (AOR = 1.999; CI = 1.076–3.715) and psychological trauma (AOR = 1.636; CI = 1.041–2.573) also shown higher odds. Dietary habits intertwined with higher odds, as daily consumers of dairy products (AOR = 1.638; CI = 1.038–2.586) and those with three (AOR = 1.813; CI = 1.21–2.717) or more daily meals (AOR = 1.891; CI = 1.2–2.979) demonstrated higher odds compared to their counterparts. Participants with moderately severe insomnia showed a more than two-fold increased oddsof FGID (AOR = 2.017; CI: 1.259–3.231) compared to those without insomnia. Moreover, individuals experiencing mild (AOR = 2.431; CI: 1.426–4.145), moderate (AOR = 2.654; CI: 1.531–4.601), moderately severe (AOR = 4.149; CI: 2.29–7.52), and severe depression (AOR = 7.017; CI: 2.739–17.975) faced elevated odds of FGID in comparison to those without depression **(Table 2)**.

**Table 2:** Logistic regression model showing the predictors of functional gastrointestinal disorder (N = 1019).

Variable name	Adjusted Odds Ratio	confidence interval	
**Gender**			
Female	Ref.		
Male	1.001	.732	1.369
**Living place**			
Home (with family)	Ref.		
Home(with relatives)	.98	.401	2.395
Hostel/mess	.974	.661	1.436
**Meal source**			
Homecooked	Ref.		
Meal from college canteen	**1.593** *	1.068	2.376
Restaurant cooked	1.119	.602	2.078
**Family income (in BDT)**			
<10000	Ref.		
10000–30000	.624	.363	1.073
30001–60000	**.432** *	.252	.74
>60000	.57	.327	.996
**Delayed meal**			
Never	Ref.		
Occasionally delayed	**1.663** *	1.031	2.682
Regularly delayed	1.872*	1.061	3.301
**Physical activity**			
Inactive	Ref.		
Moderately active	.899	.626	1.291
Very active	**.409** *	1.061	3.301
**Family history**			
None	Ref.		
Functional gastrointestinal disease	**4.697** *	2.547	8.662
Other Gastrointestinal disease	2.417*	1.466	3.985
Other diseases	1.943*	1.337	2.823
**History of abdominal surgery**			
No	Ref.		
Yes	1.999*	1.076	3.715
**History of psychological trauma**			
No	Ref.		
Yes	1.636*	1.041	2.573
**BMI**			
Underweight	Ref.		
Normal	1.641	.991	2.718
Overweight/Obese	1.608	.936	2.762
**Fast-food intake**			
Never	Ref.		
1–3 times/week	.849	.581	1.242
Daily	.900	.543	1.491
**Fruits and vegetable intake**			
Never	Ref.		
1–3 times/week	.662	.328	1.337
Daily	.590	.285	1.222
**Dairy products intake**			
Never	Ref.		
1–3 times/week	1.101	.767	1.579
Daily	**1.638** *	1.038	2.586
**Caffeine intake**			
Never	Ref.		
1–3 times/week	.752	.475	1.19
Daily	.750	.425	1.323
**Number of meals per day**			
≤ 2 meals/day	Ref.		
3 meals/day	1.813*	1.21	2.717
>3 meals/day	1.891*	1.2	2.979
**Insomnia**			
No insomnia	Ref.		
Subthreshold insomnia	1.391	.969	1.995
Moderately severe insomnia	**2.017** *	1.259	3.231
Severe insomnia	1.984	.729	5.395
**Stress**	1.039	.975	1.107
**Depression**			
Minimal depression	Ref.		
Mild depression	2.431*	1.426	4.145
Moderate depression	2.654*	1.531	4.601
Moderately severe depression	4.149*	2.29	7.52
Severe depression	7.017*	2.739	17.975
**Screen addiction**			
Not addicted	Ref.		
High risk	.717	.277	1.857
Addicted	.813	.333	1.98

* p-value<0.05.

## Discussion

FGIDs pose a pressing global health concern, predominantly affecting young adults. These conditions comprise a range of gastrointestinal issues marked by distressing symptoms, constituting a significant challenge to public health [39]. Despite their recognized global impact, a noticeable gap exists in comprehensive cross-sectional studies evaluating FGID’s prevalence and associated factors, particularly among undergraduate students in Bangladesh. Our study aimed to address this knowledge gap by investigating FGID prevalence and its predictors among this demographic, offering essential insights into their digestive health.

The current study revealed that approximately 38.24% of undergraduate students in Bangladesh experienced symptoms indicative of FGID. This finding strongly resonates with the mounting global concern surrounding the prevalence of FGID among young adults [40–44]. FGID encompasses a range of disorders, often characterized by symptoms such as abdominal pain, bloating, and altered bowel habits, which impact a substantial portion of this demographic. Notably, Goyal et al. conducted a study in India using Rome IV criteria, which reported a similar prevalence of FGID among college students, reinforcing the consistency of our study’s findings [14,45].

Within the spectrum of FGIDs, constipation emerges as a prominent issue, with a significant number of students in our study reporting constipation-related symptoms. This finding aligns with prior research conducted by Ribas et al. in Catalonia, Spain, which reported a substantial prevalence of functional constipation (24.2%) within the general population [46]. Additionally, Hisham et al. documented that as children in Makkah City aged, they experienced functional constipation and stomach pain more frequently, with constipation being notably more common than functional abdominal pain [47]. This age-related trend in constipation prevalence corresponds to our study’s observations, suggesting that the burden of functional constipation may increase among young adults in Bangladesh.

A noteworthy observation from our study was that students who relied on college canteen meals faced a higher likelihood of FGID, raising concerns about dietary quality and hygiene in educational institutions. This finding aligns with Malik et al.’s study, which found a significant association between dining out and epigastric pain, emphasizing the relevance of meal choices in gastrointestinal health [48]. Furthermore, Shau et al. established an association between fast food consumption and FGID among adolescents, further reinforcing the importance of dietary choices in our study’s context [49]. These findings collectively underscored the importance of promoting healthier dietary practices within and outside educational institutions to address FGID risk factors.

The study underscored the significance of meal timing for digestive health, as we observed that occasional and regular delayed meal patterns were associated with an elevated likelihood of FGID among the participants. This finding aligns with research conducted by Yamamoto et al., who identified an independent positive correlation between delayed meal patterns and Functional Dyspepsia (FD) among Japanese university students [50]. Similarly, Mullan et al. conducted a European survey, revealing associations between unpredictable eating habits, eating hurriedly, and FD [51] Furthermore, our findings are consistent with the work of Agah et al. and Keshteli et al., emphasizing similar associations and highlighting the importance of maintaining regular meal schedules for promoting gastrointestinal well-being [52,53].

A significant finding in our study pertained to the link between daily consumption of dairy products and an increased likelihood of FGID, in addition to the practice of having three or more meals per day. These dietary patterns carry significant implications for digestive health among our participants. Interestingly, this finding contrasts with some prior research, as demonstrated by Bouzid et al.’s and Moosavian et al.’s study, which found no substantial association between dairy consumption and FGID [54,55]. These disparities underscore the intricate and variable nature of the relationships between dietary factors and FGIDs, emphasizing the imperative for more comprehensive investigations to fully elucidate these complexities in light of our study’s findings.

The interplay between physical activity and FGIDs emerged as a key focus in our study, emphasizing the significant influence of lifestyle on digestive health. Our data revealed a compelling association between individuals with lower physical activity levels, including those who led sedentary lifestyles or had limited exercise routines, and significantly elevated odds of developing FGIDs. These findings aligned with the studies by Bi et al. [56] and Sadeghian et al. [57], emphasizing the necessity of promoting active living as a vital component of preventive healthcare strategies and providing further validation for our study’s results.

In our study, we observed a convincing relationship between family history and the development of FGID, which aligned with the findings of several prior studies. Morris-Yates et al. [58] and Ruderstam et al. [59] had previously reported a familial pattern suggesting potential genetic predisposition to FGIDs and heritability of functional bowel disorders. Moreover, Levy et al. [60] highlighted the role of gene polymorphisms in the pathophysiology of FGIDs in families or twins, which further supported the notion of a genetic component contributing to these disorders. These collective findings emphasize the role of genetic and familial factors in developing FGIDs, shedding light on potential avenues for further genetic research and clinical interventions.

The research findings indicated a significant correlation between individuals with a history of abdominal surgery and the likelihood of experiencing FGIDs. This aligns with prior research conducted by Kim et al. [61] and Sung et al. [62], which also found a connection between previous surgical experiences and gastrointestinal discomfort. Additionally, a strong association was observed between a history of psychological trauma and gastrointestinal health, consistent with previous studies showing heightened pain responsiveness in chronic pain patients with trauma histories [63–65]. These results underscore the importance of considering surgical and psychological histories when addressing FGIDs, emphasizing the need for comprehensive healthcare approaches.

The study also highlighted that participants with insomnia and varying levels of depression (mild, moderate, and moderately severe) had an elevated risk of FGIDs, which reinforces the intricate connection between mental health and gastrointestinal well-being, consistent with prior research [66–69]. Moreover, we observed a suggestive link between screen addiction and FGIDs, with students categorized as ’addicted’ to screens displaying a higher prevalence of FGIDs. Interestingly, this finding contradicts the research conducted by Samuel et al. [70] and necessitates further exploration to unravel the complex relationship between screen time and gastrointestinal health.

## Limitations

It’s crucial to acknowledge certain limitations in our study, which centers around the prevalence and predictors of FGID. Since all the data came from self-reported responses, there’s a possibility that recall bias and individual interpretations might have influenced the outcomes. Furthermore, the quantitative nature of our research might not fully capture the nuanced perspectives of our participants and the cross-sectional design limits exploring changes over time. Due to limitations in time and funding, we resorted to non-probability sampling, which could introduce selection bias into our sample. Moreover, the sample consisting solely of undergraduate students in Bangladesh limits the generalizability of the findings to other populations. Despite limitations, this is the first study among Bangladeshi undergraduate students which examined the prevalence and predictors of FGIDs. Also, the large sample size in this study helped to portray the true representation of this ongoing issue. Nonetheless, to comprehensively address these aspects related to FGID prevalence and predictors, future research should consider a longitudinal design with a larger and randomly selected sample.

## Conclusion

Our study elucidated the burden of FGIDs among Bangladeshi undergraduate students. We found significant factors contributing to their prevalence, including meal source and number of daily meals, delayed meals, family history of disease, physical activity, abdominal surgery, history of psychological trauma, depression, and insomnia. While our findings align with previous research, they emphasize the need for further exploration and holistic healthcare approaches to better the well-being of this vulnerable population worldwide dealing with FGIDs.

## Supporting information

S1 Appendix Dataset. (XLSX)
